# A schema for sporadic and heritable disease pathogenesis integrating spatiotemporal distribution with the character of genetic variants

**DOI:** 10.1038/s43856-025-01039-7

**Published:** 2025-08-08

**Authors:** Hussam Alkaissi, Yasemin Cole, Tara T. Doucet-O’Hare, Jared S. Rosenblum, Zhengping Zhuang, Karel Pacak

**Affiliations:** 1 National Institute of Diabetes and Digestive and Kidney Diseases, National Institutes of Health, Bethesda, MD 20892 USA; 2 Neuro-Oncology Branch, National Cancer Institute, National Institutes of Health, Bethesda, MD 20892 USA; 3 Center for Human Genetics, Clemson University, Greenwood, SC 29646 USA; 4 Department of Genetics and Biochemistry, Clemson University, Clemson, SC 29631 USA; 5 Section on Medical Neuroendocrinology, Eunice Kennedy Shriver National Institute of Child Health and Human Development, National Institutes of Health, Bethesda, MD 20892 USA; 6 Center for Adrenal Endocrine Tumors, AKESO, 158 00 Prague 5, Czech Republic

**Keywords:** Cancer genetics, Endocrine cancer

## Abstract

Alkassi, Cole et al. provide a conceptual framework for understanding how the molecular characteristics and spatiotemporal distribution of HIF2α variants impact the disease phenotype in Pacak–Zhuang Syndrome. This schema has application not only to this syndrome, but also hereditary cancer syndromes and other mosaic diseases.

## Teaser

Studies on HIF-2α variants have shed light on genotype-phenotype correlations in Pacak-Zhuang syndrome. Here, we propose the combination of the timing of variant acquisition and molecular pathogenicity into a schema to understand phenotypic severity, with implications for other diseases.

## Introduction

The interest in hypoxia-inducible factors (HIFs) and their role in biology and the pathophysiology of various diseases has grown significantly over the past three decades, particularly following the discovery of *VHL* gene variants in Von Hippel–Lindau (VHL) disease, which is a hereditary tumor predisposition syndrome characterized by neuroendocrine tumors, including paraganglioma/pheochromocytoma (PPGL)^1–3^. Building on this foundation, we identified somatic variants in *HIF2A* (*EPAS1*) in multiple individuals with a syndrome of a triad of PPGL, polycythemia, and somatostatinoma, also known as Pacak–Zhuang syndrome (PZS)^4^. Since their initial discovery in VHL disease^5^, HIFs have long been recognized as critical mediators of homeostasis and biological processes, including angiogenesis, proliferation, and migration^2^. In the presence of oxygen, HIF-α proteins are hydroxylated by prolyl-hydroxylase domain-containing proteins (PHD), then ubiquitinated by VHL complex and degraded by the proteosome^2^. In response to hypoxia, HIF-α proteins are stabilized and dimerize with HIF-ß, and the complex travels to the nucleus, binding to hypoxia response elements (HRE) across the genome, altering gene expression. The discovery of *HIF2A* pathogenic variants not only explained the syndromic manifestation but also established the first direct causative link between hypoxia signaling and cancer biology^4,6^. Since the initial findings, we have continued to investigate this syndrome, expanding our understanding of the role of HIF-2α in clinically observed neurodevelopmental and vascular anomalies. Moreover, the investigation of HIF-2α in PPGL pathophysiology catapulted efforts to molecularly stratify these tumors into three main clusters: Cluster 1—pseudohypoxia cluster— related to HIF-2α and Krebs cycle defect, Cluster 2 related to increased kinase activity, and Cluster 3 related to the Wnt signaling pathway.

## Genetic variant character and disease phenotype

Recently, Ferens et al. proposed a genotype-phenotype classification of PZS based on the structural biochemistry of HIF-2α variants and the correlated phenotypes^7^. Using wild-type and mutant synthetic proteins and microscale thermophoresis to measure protein-protein interactions, Ferens et al. demonstrated that HIF-2α variants affect the affinity of HIF-2α for binding to prolyl hydroxylase-2 (PHD2)^7^. The authors suggested that major alterations in the oxygen-dependent degradation (ODD) domain of HIF-2α would result in very low to no binding affinity to PHD2, resulting in more stable HIF-2α, and thus manifesting as the full disease triad of PZS^7^. Alternatively, less disruptive HIF-2α variants with moderate effect on the affinity of HIF-2α for PHD2 would manifest as only polycythemia (elevated red blood cell count). This is the first clinically relevant classification schema explaining the variability in the clinical phenotypes of patients. This represents an important milestone in the field, relating to our continuously evolving understanding of disease pathogenesis with disease phenotype, as did the molecular stratification of PPGLs into 3 clusters^8,9^. Our phenotyping studies of PZS have suggested that molecular stratification alone may not be the only consideration to explain disease extent, as the timing of acquiring these variants in development, e.g., early vs late somatic mosaicism (as defined below), may be as important as the class of variant in determining the disease phenotype, as evidenced by the varying allele frequencies throughout multiple tissues in our patients^10^. Whether there are other contributing factors, including acute or prolonged oxygen oscillations *in utero*, geographical location (especially areas of high altitude), or some specific environmental factors, including endocrine-disrupting chemicals, should be strongly considered and warrant further investigation.

## Genetic variant spatiotemporal distribution and disease phenotype

The precise location and character of genetic variants are generally believed to be a critical determinant of disease phenotype in germline neoplastic syndromes^11,12^. For example, in VHL disease, which shares a molecular pathogenic pathway of increased hypoxia signaling with PZS, the location and type of pathogenic variants within the *VHL* gene contribute critically to phenotypic presentation^13^. Germline missense and truncating pathogenic variants with later somatic loss of heterozygosity (LOH) lead to a predisposition of hemangioblastomas, PPGLs, and renal cell carcinoma (RCC), i.e., VHL disease^13^. However, the biallelic c.598 C > T pathogenic variant in the same gene (3′ to the classic VHL-associated pathogenic variants) results in a different phenotype—polycythemia and congenital vascular anomalies in a syndrome known as Chuvash polycythemia^14^. This underscores the impact of the type and structural biochemistry of the *VHL* genetic variant on the resultant disease phenotype.

The discovery of PZS, which has a subtly different disease phenotype to VHL disease, provided the first direct evidence that aberrant hypoxia signaling mediated by HIF-2α is sufficient to cause tumor development. While VHL disease is caused by a germline variant and, therefore, may potentially affect every cell throughout the body, PZS is caused by somatic mosaicism with variable allele frequency, or degree of mosaicism, throughout the soma^10^. This insight led to our understanding that the absence of certain phenotypic features in patients with neoplastic syndromes may result from the absence, or variable frequency, of the variant in a given tissue, rather than purely the type of genetic variant, which determines the strength of association between HIF-2α and PHD2. Taken together, the insights from the study by Ferens et al.^7^ and our previous work suggest that to understand the phenotypic development in neoplastic syndromes, the structural biochemistry of a genetic variant and its distribution in the soma would need to be combined.

## Illustrative cases

**Fig. 1:**
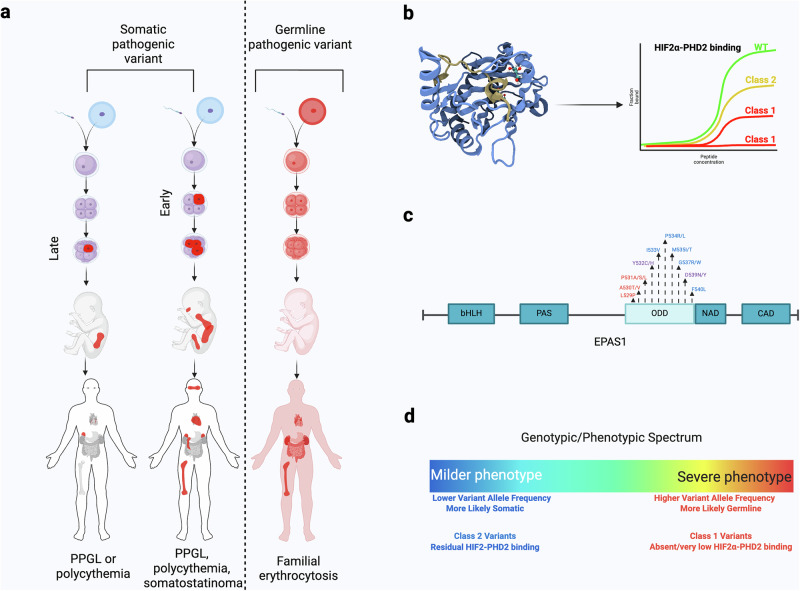
Proposed molecular genetic schema to explain disease presentation in Pacak–Zhuang syndrome (PZS). Incorporation of multiple levels of molecular and genetic information enables the understanding of the phenotypic spectrum of PZS. **a** Diagram of possible disease outcomes resulting from differences in the spatiotemporal distribution of genetic variants in *EPAS1*, which encodes hypoxia-inducible factor 2α (HIF-2α). We previously found that somatic mosaicism in HIF-2α results in PZS with varying phenotypes, while others previously found that germline variants in *EPAS1* may result in familial erythrocytosis. **b** Three-dimensional structure of the PHD2 protein (light blue) bound to the oxygen degradation domain (ODD) of HIF-2α (tan) (PDB ID: 7UJV). This domain contains mutational hotspots in HIF-2α that alter its binding affinity to PHD2, forming the basis of ‘class variants’ described by Ferens et al.^1^. Class 1 variants exhibit the weakest binding affinity, while class 2 variants bind more strongly than class 1 but still less than wild-type HIF-2α. **c** A graphical representation of genetic variants in *EPAS1* and their classification according to Ferens et al. is shown: class 1 variants in red, class 2 in blue, and residues such as Y532 and D539 that may result in either class 1 or 2 in purple. **d** We propose an expansion of the Ferens et al. framework to include the spatiotemporal distribution of *EPAS1* variants, based on its allele frequency across multiple tissues. Variants with higher allele frequencies are associated with more severe phenotypes, reflecting earlier acquisition, ranging from germline to later somatic events. We further suggest that this distributional component can be integrated with the structural biochemistry classification defined by Ferens et al. Created in BioRender. Alkaissi, H. (2025) https://BioRender.com/f30algg.

Two individuals from our cohort illustrate this point. First, an individual with PZS (Patient 1) presented with a mild phenotype—no somatostatinoma, polycythemia, and no evidence of PGL recurrence after resection of multiple tumors within the last six years. We would expect the genetic variant (*EPAS1*^*P531S*^*)* in this individual to result in a more severe phenotype based on the predicted loss of interaction between PHD2 and HIF-2α^7^. However, the phenotype was mild, which we believe is explained by the lower degree of mosaicism in multiple tissues (Table 1). In contrast, another individual in our cohort (Patient 2) presented with a more severe clinical course with recurrence of PGLs after four surgeries, frequent catecholaminergic crises, and a metastatic somatostatinoma (Table 1). This individual, notably, had a high variant allele frequency (25%) of *EPAS1*^A530T^, which we would expect to produce a milder phenotype according to the study by Ferens et al. These cases highlight the importance of considering the degree of mosaicism, i.e., the spatiotemporal distribution of acquired genetic variants, in disease classification. Clinical phenotypes fall on a spectrum from high to low mosaicism, following different natural histories and, therefore, may require more individualized clinical care, as reflected in our schema (Fig. 1).

**Table 1:** Pacak–Zhuang patient characteristics

Pacak–Zhuang syndrome patient characteristics		
	Patient 1	Patient 2
*Phenotype*		
Polycythemia		
Age of Initial Diagnosis (years)	3	0
RBC Count (3.905.2 M/µL)	6	9.2
Erythropoietin (2.6–18.5 mIU/mL)	42.4	471
Somatostatinoma		
Age of Initial Diagnosis (years)	No somatostatinoma	29
Somatostatin (<25 pg/mL)	13	109
Age of Recurrence/Metastatic Disease (years)	–	31
Paraganglioma		
Age of Initial Diagnosis (years)	31	14
Age of Recurrence/Metastatic Disease	31.5, 35	23, 29, 32, 34, 36
Disease-Free Survival (years)	6	–
Ocular Malformations		
Age of Initial Diagnosis (years)	31	32
Visual Acuity	OD 20/20; OS 20/20	OD 20/25; OS 20/25
Congenital Optic Disc Anomaly	No	Yes
Fibrovascular Membrane/gliosis	No	Yes
Hemangiomatous Lesions	Yes	Yes
Telangiectatic Lesions	No	Yes
Laboratory Investigations		
*Catecholamines (plasma)*		
Norepinephrine (80–498 pg/mL)	1026	10951
Epinephrine (4.0–83 pg/mL)	<20	100
Dopamine (3.0–46 pg/mL)	41	28
Normetanephrine (18–112 pg/mL)	688	4834
Metanephrine (12.0–61 pg/mL)	<12	121
Variant analysis		
*EPAS1*	p. P531S	p. A530T
Predicted Km (µM) [1]	No binding	627 ± 126 (18×)
Variant allele frequency (%):		
Blood	Undetected	12.73%
Hair	2.30%	12%
Tumor	24.5% (adrenal)	56.5% (perinephric mass)

Clinical phenotypes and laboratory investigations are given for two individuals with Pacak–Zhuang syndrome from our cohort to highlight the difference in disease presentation that reflects the impact of both structural biochemistry and spatiotemporal distribution of the acquired genetic variant. Age of onset is provided for each phenotype. For tumors such as somatostatinoma and paraganglioma, information regarding disease recurrence is provided. Clinical phenotypes have been published previously^19–21^. Patient 1 had a catecholamine crisis, which resolved post-surgery. Patient 2 continued to experience catecholamine crises, despite surgery. M: molar; µL: microliter; mIU/mL: milli-international units per milliliter; pg/mL: picograms per milliliter; µM: micromolar.

Several neoplastic syndromes have a variable presentation and natural history, even in individuals harboring the same variant, as well as within individuals in families with hereditary cancer syndromes^15^. For example, germline pathogenic variants in neurofibromin 1 (*NF1*), lead to neurofibromatosis, a tumor predisposition syndrome characterized by 100% penetrance of systemic disease by age 5, while somatic and/or mosaic pathogenic variants in *NF1* result in isolated neurofibromas with later onset^16^. Further, even within the same disease, e.g., germline neurofibromatosis (both de novo and hereditary), the disease phenotype may present differently based on the spatiotemporal distribution of the expression of the altered gene^16^, which can be mediated by the tissue specificity of the gene expression and parent-of-origin imprinting, and, therefore, may affect some tissues more than others^16,17^. Our schema may explain other diseases with tissue-restricted phenotypes. While our proposed schema considers the type and spatiotemporal distribution of the pathogenic variant, which likely explains most disease pathogenesis, factors that modify the expression of an acquired variant may also alter disease presentation. For example, a recent study including a remarkable international collaboration between European and South American centers reported significant phenotypic differences, such as age of onset or laterality, in patients with neuroendocrine tumors due to the same pathogenic variants^18^. Bilateral pheochromocytomas were more common in patients from Europe, as compared to South American patients, and were more likely to present the disease at an earlier age.

## Concluding remarks

In summary, incorporating the degree of somatic mosaicism and the spatiotemporal distribution of pathogenic variants would be as important as identifying the pathogenic variant and its effect on protein functions and interactions. This would apply to PZS and, more broadly, to known diseases, including tumor predisposition syndromes and sporadic disease. With this new classification, patients with localized (late mosaicism) pathogenic variants would have a milder and more localized clinical manifestation even if the variant is molecularly classified as more deleterious, and vice versa.

### Reporting summary

Further information on research design is available in the Nature Portfolio Reporting Summary linked to this article.

## Supplementary information

Peer Review file

Reporting Summary
